# The negligible mutagenic effects of norfloxacin on the genome of the fission yeast *Schizosaccharomyces pombe* ATCC-16979

**DOI:** 10.1128/spectrum.00233-25

**Published:** 2025-07-25

**Authors:** Tongtong Lin, Xiaolin Wu, Yelin Lan, Yongchun Huang, Ziguang Deng, Yu Zhang, Michael Lynch, Hongan Long, Jiao Pan

**Affiliations:** 1 Key Laboratory of Evolution and Marine Biodiversity (Ministry of Education), Institute of Evolution and Marine Biodiversity, Ocean University of China 535359, Qingdao, Shandong Province, China; 2 Laboratory for Marine Biology and Biotechnology, Laoshan Laboratory 474988, Qingdao, Shandong Province, China; 3 School of Mathematics Science, Ocean University of China 12591, Qingdao, Shandong Province, China; 4 Biodesign Center for Mechanisms of Evolution, Arizona State University 7864, Tempe, Arizona, USA; University of Wisconsin-Madison, Madison, Wisconsin, USA

**Keywords:** antibiotic-induced mutagenesis, mutation accumulation, mutations, evolution

## Abstract

Antibiotic therapy is commonly used in various medical scenarios, and some antibiotics will stay in the blood serum for days. Previous studies have demonstrated that the fluoroquinolone antibiotic norfloxacin exerts mutagenic effects on the whole genomes of target bacteria. However, whether and to what degree these effects compromise non-target eukaryotic genomes remains unclear. Here, we explored this using mutation accumulation experiments with the fission yeast *Schizosaccharomyces pombe* ATCC-16979, treated with or without norfloxacin at a dose comparable to the therapeutic concentration found in patient blood. Based on a *de novo* assembly and the mutation accumulation lines without treatment, ATCC-16979 demonstrates a strong mutation bias in the A/T direction (4.33). Furthermore, norfloxacin treatment did not significantly elevate the genomic mutation rate of the fission yeast, based on the analysis of 94 mutation accumulation lines run for ~169,000 cell divisions in total. Nucleotide excision repair was induced by the norfloxacin treatment and might help to counteract the possible mutagenic effects of norfloxacin, as revealed by RNAseq-based differential gene-expression analyses. Norfloxacin thus poses a negligible genotoxic threat to eukaryotic and potentially human genomes, bolstering the safe clinical use of this important antibiotic.

**IMPORTANCE:**

This study addresses concerns about the potential mutagenic side effects of antibiotics, specifically norfloxacin, which is widely used in clinical settings. While previous research has shown that norfloxacin can cause mutations in bacteria, it was unclear whether it could also harm human or other eukaryotic genomes. By using the fission yeast *Schizosaccharomyces pombe* as a model, we found that norfloxacin treatment did not significantly increase the mutation rate in eukaryotic cells, possibly resulting from a cellular repair mechanism counteracting potential DNA damage. These findings provide reassurance that, at therapeutic levels, norfloxacin does not pose a significant genetic risk to eukaryotic organisms, supporting its continued safe use in medical treatments.

## INTRODUCTION

Over the past few decades, antibiotics have been extensively used in agriculture, aquaculture, and animal husbandry for disease treatment, growth promotion, and prophylaxis (1–3). Prophylactic antibiotics in shrimp and salmon farming have disseminated widely in aquatic ecosystems, including rivers, lakes, and oceans (1, 4). This widespread use of antibiotics in food animals has led to escalating bacterial resistance in the environment and its transmission to human populations (5–7). Mutational events drive antibiotic resistance in bacteria, and resistance phenotypes may increase with antibiotic concentration (8–11). However, few studies have explored the relationship between antibiotics and mutation rates within non-target eukaryotic genomes.

Antibiotics are commonly used in clinics to treat bacterial infections caused by various pathogens, such as *Escherichia coli*, *Streptococcus pneumoniae*, and *Salmonella*, and often affect the mutations of these bacteria (12–14). Typical examples of these infections include bacterial cold, gastroenteritis, otitis media, and bacterial pneumonia. Norfloxacin is one of the fluoroquinolone antibiotics that is widely used in clinical settings, and it is effective against most gram-negative pathogens, including *E. coli* and certain species of *Klebsiella*, *Enterobacter*, *Proteus*, and *Citrobacter* (15). Norfloxacin affects bacterial DNA replication by inhibiting the subunit of the essential enzyme DNA gyrase, leading to the formation of crosslinked protein-DNA complexes and DNA breaks, which subsequently induce the SOS response (16, 17). Our previous studies have demonstrated a linear correlation between the mutation rates of *E. coli* and norfloxacin dosage, as well as the expression levels of low-fidelity DNA polymerases in the SOS response pathway (10). Furthermore, we have previously confirmed that the SOS response is the primary contributor to mutagenesis in *E. coli*, responsible for approximately 30%–50% of the total base-pair substitution (BPS) mutation-rate increase upon exposure to sublethal levels of norfloxacin (0–50 ng/mL) (18). The therapeutic application of norfloxacin has been shown to be effective in the primary prevention of spontaneous bacterial peritonitis and to improve survival rates in individuals with cirrhosis (19–21).

While antibiotics provide significant benefits in curing diseases, they also pose a looming threat to humans. In addition to having the potential to induce bacterial resistance and compromise the effectiveness of future treatments, residual antibiotics in the human body after treatment raise concerns for human cells. Norfloxacin, for example, has shown efficacy in treating urinary cystitis. However, it is important to acknowledge the potential risk of norfloxacin-induced crystalluria in humans, as norfloxacin is present in urine and serum (22, 23). In adults receiving norfloxacin, the mean peak concentration in blood serum is typically reached within 1 to 2 hours after drug administration (24). To enhance the clinical safety of antibiotics, it is crucial to understand the impact of antibiotic persistence in serum on human cell function.

The fission yeast *Schizosaccharomyces pombe* serves as an invaluable model for studying gene function, given that nearly half of the genes are homologous to those in humans and exhibit highly conserved functions (25, 26). *S. pombe* is widely recognized and extensively used in biological investigations. Notably, several homologous genes found in *S. pombe*, such as *btn1*, *rad1*, *hus1*, and *rad9*, have been implicated in human diseases (27, 28). Moreover, the *S. pombe* genome harbors 23 genes similar to human cancer-related genes, playing critical roles in essential processes that maintain genomic stability, including DNA damage response, repair mechanisms, checkpoint controls, and related pathways (29). These findings underscore the significance of utilizing *S. pombe* as a valuable model system for investigating and comprehending disease states. Consequently, *S. pombe* becomes an ideal candidate for exploring the mutagenic effects of antibiotics on the eukaryotic genome.

In this study, we conducted mutation accumulation (MA) experiments on the wild-type strain *S. pombe* ATCC-16979, by repeatedly bottlenecking of dozens of cell lines through single-colony transfers over thousands of generations and the treatment of different norfloxacin doses (0 and 4 µg/mL). This approach allowed drift to dominate selection, leading to the accumulation of various mutations, even those that are largely deleterious (30, 31), and used deep whole-genome sequencing to assess the mutagenic effects of residual norfloxacin on the eukaryotic genome. Prior to this, we performed high-quality *de novo* assembly of the *S. pombe* ATCC-16979 strain using Oxford Nanopore long-read sequencing combined with Illumina PE150 sequencing, followed by a comparative genomic analysis with the type strain *S. pombe* 972h-. We also did RNAseq-based differential gene-expression analyses to explore the mutagenesis mechanisms induced by norfloxacin treatment. The integration of these findings allowed us to make informed inferences concerning the potential genotoxic effects of norfloxacin on the eukaryotic genome.

## MATERIALS AND METHODS

### Strain and media

The wild-type *S. pombe* ATCC-16979 was ordered through the Query Network for Microbial Species of China (http://www.biobw.org/; catalog number: bio-58021) and identified using 26S rRNA partial sequence provided by ATCC. Yeast extract Peptone Dextrose medium (YPD) agar plates (Cat No.: LA0220, Solarbio) and YPD broth (Cat No.: LA5010, Solarbio) were used for culturing *S. pombe* ATCC-16979. Stock solutions of norfloxacin (Cat No.: SN8340, Solarbio) were made according to the manufacturers’ instructions.

### MA experiments

In order to reveal the genomic mutation rate and spectrum in *S. pombe* ATCC-16979 under antibiotic pressure, we ran MA experiments—the most accurate method for mutation rate estimation to date—on a wild-type strain *S. pombe* ATCC-16979, following the protocols by Farlow et al. (32). Briefly, we created two groups of MA lines from a *S. pombe* ATCC-16979 ancestral single colony, with one group of 100 MA lines cultured on YPD agar plates (labeled as control) and the other group of 100 MA lines on YPD agar plates supplemented with 4 µg/mL norfloxacin (labeled as treatment), which is close to the norfloxacin concentration in serum two hours after a patient takes a 1,600 mg tablet (24). The single colony of each MA line was transferred every three days for approximately 46 times on average.

About every ten transfers, we estimated cell divisions that the MA lines had passed by colony forming units (CFU) of diluted single colonies from 10 randomly selected MA lines for each group. The total cell division number of each MA line is the product of the grand mean of all cell division estimates and the total transfers of each line. The effective population size (*Ne*) was calculated by a harmonic mean method (33), where *Ne* = $\frac{T+1}{\sum_{i=0}^{T}\frac{1}{\;2^{i}}}$, where *T* is the number of divisions between transfers.

### Genomic DNA extraction, library construction, and genome sequencing

For *de novo* assembly, we extracted genomic DNA of ancestral *S. pombe* ATCC-16979 using the CTAB DNA extraction method, after which the purity and concentration of the resultant DNA were assessed using the Nano-300 spectrophotometer (Allsheng, Hangzhou, China) and the Qubit 3.0 Fluorometer (Life Technologies, Carlsbad, CA, USA). DNA libraries were constructed using the Oxford Nanopore Technologies LSK109 library preparation kits following the manufacturer’s instructions. They were sequenced on an R9.4 flow cell with an Oxford Nanopore PromethION machine by Benagen (Wuhan, China). We also sequenced the ancestral *S. pombe* ATCC-16979 for Illumina PE150 sequencing. The DNA was prepared with the Yeast DNA Kit (OMEGA No.: D3370), and the DNA library was constructed by following Li et al. (34) with insert size around 300 bp. Illumina Novaseq 6000 sequencing was then done by Berry Genomics, Beijing, China.

Genomic DNA of *S. pombe* ATCC-16979 final MA lines was prepared with the Yeast DNA Kit (OMEGA No.: D3370). For cost concerns, we chose 50 samples from each group and constructed DNA libraries by following Li et al. (34) with 300 bp insert size, and Illumina Novaseq 6000 sequencing was then done by Berry Genomics, Beijing, China.

### Genome assembly and annotation of *S. pombe* ATCC-16979

Nanopore sequencing yielded 183,031 raw reads, ~2.24 Gbp with the longest read of 159,835 bp for *S. pombe* ATCC-16979. Raw Nanopore data were filtered by NanoFilt (35), and reads with quality scores below 8 and sequence lengths less than 1,000 bp were filtered out. After filtering, 2.12 Gbp of Nanopore reads were retained, and then, we used Flye (v-2.9) (36) for long-read assembly with parameter “-asm-coverage = 50”. Then, in order to improve the quality of the draft genome, the assembly was corrected for error bases, misassemblies, and gaps by Pilon (v-1.23) (37), using 2.03 Gbp Illumina PE150 reads of the ancestral *S. pombe* ATCC-16979, with default parameters. Genome assembly was assessed by Quast (v-5.2.0) (38) and BUSCO (v-5.4.3) (39). Transcriptome assembling was carried out with Trinity (v-2.12.0) (40) using default parameters, and the genome annotation was done by Augustus (v-3.5) (41), Braker2 (v-5.1.1) (42), and OmicsBox (v-1.4.11) (43). tRNA was identified by tRNAscan-SE (v-2.0.9) (44).

### Mutation analyses and statistics

For paired-end raw reads of all final MA lines, we used Trimmomatic (v-0.38) to trim off adaptors and filter out low-quality data and then mapped the clean reads to the reference genome (GWHBPBN00000000, China National Center for Bioinformation), using Burrows-Wheeler Aligner (v-0.7.17) MEM (45, 46). Duplicate reads were removed using picard-tools (v-2.17.2). BPSs and indels (insertion-deletion mutations) were discovered using standard hard filtering parameters described by the HaplotypeCaller module of GATK (v-4.1.2) (47, 48). Mutation curation was done using IGV (v-2.8.12) (49).

All statistics and illustrations were done with R packages ggplot2, ggpubr, dplyr, forcats, gridExtra, viridis, and MASS in R (v-4.0.2) (50).

### RNAseq of *S. pombe* ATCC-16979

To explore mutagenesis mechanisms at the gene-expression level involved in the treatment, we performed RNAseq-based differential gene-expression analyses. For RNAseq of *S. pombe* ATCC-16979, colonies were grown at the same condition as MA. From a single ancestral colony of *S. pombe* ATCC-16979, we first streaked it onto YPD agar plates and cultured for three days at 30°C. Single colonies were then streaked onto four YPD agar plates (control) and another four YPD agar plates supplemented with 4 µg/mL norfloxacin (treatment). After three days of incubation at 30°C, cells on each plate were then washed down with 1.8 mL cold sterilized water and transferred to 2.0 mL Eppendorf tubes. Total RNA was then extracted using the Yeast RNA Kit (OMEGA Cat No.: R6870-01). The concentration and purity of the RNA were measured with a Qubit 3.0 fluorometer (Life Technologies, Carlsbad, CA, USA) and a micro-volume spectrophotometer instrument (Nano-300), respectively. Sequencing libraries were generated using the NEBNext Ultra RNA Library Prep Kit for Illumina (NEB, USA) and then sequenced on an Illumina Novaseq 6000 platform with PE150 mode at Berry Genomics, Beijing, China.

### Differential gene expression and pathway enrichment analyses of *S. pombe* ATCC-16979

For RNAseq data of *S. pombe* ATCC-16979, clean reads were obtained by removing reads containing adaptors, reads containing ploy–N and low-quality reads from raw data using fastp (v-0.20.0) (51). The reference genome was indexed using Hisat2 (v-2.1.0), and then paired-end clean reads were mapped to the reference genome (52). Next, sam files were sorted and converted to bam files by SAMtools (v-1.9) (53). Then we used StringTie (v-1.3.7) (54) to assemble the transcriptome and predict new genes. Differential expression genes (DEGs) analysis of the two conditions was performed using the DESeq2 R package (1.16.1) (55). The construction of the GO term database and GO enrichment analysis were performed using clusterProfiler (v-4.0) (56). Gene enrichment analyses and functional annotation were achieved by DAVID (v2023q1) (57).

## RESULTS

### Genome assembly of *S. pombe* ATCC-16979

For precise mutation calling and differential gene-expression analyses, we first *de novo* assembled the reference genome of *S. pombe* ATCC-16979.

The assembly of ATCC-16979 contains five contigs. The genome size is 12,521,788 bp (GC content 36.06%) (Table 1). There are 5,578 predicted protein-coding genes, of which 2,729 are orthologous to human genes (47.55% vs 51.72% in the type strain 972h-) and 183 tRNAs (Table 1). Meanwhile, 97.56% of the protein-coding genes in ATCC-16979 are orthologous to those in 972h-, accounting for 93.00% of the protein-coding genes in 972h-.

**TABLE 1:** Summary of the assembly and annotation of *S. pombe* ATCC-16979^*a*^

Genomic features	*S. pombe* ATCC-16979
Contigs	5
Largest contig	5,563,033 bp
Total length	12,521,788 bp
GC (%)	36.06
N50	4,494,244 bp
N per 100 kb	0
BUSCO score	79.4%
Predicted genes	5,578
Number of tRNAs	183
Orthologous genes to human	2,651

^*a*^ N, the number of gaps in the assembly.

### High norfloxacin concentration does not increase the genomic mutation rate of *S. pombe* ATCC-16979

**Fig 1:**
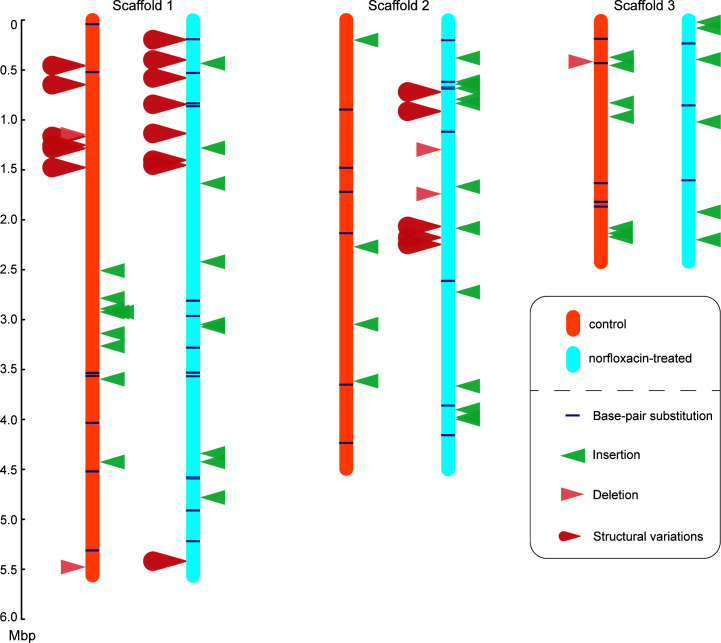
Distribution of different types of mutations across different scaffolds in the control vs norfloxacin-treated MA lines. Note that two tiny scaffolds without any mutation hits are neglected from this figure.

100 MA lines were initiated on YPD plates for each group, with each line experiencing 890 and 911 cell divisions on average, for control and treatment, respectively. 50 lines (even-numbered lines were chosen to avoid cross-contamination between two lines on the same Petri dish) of each group were constructed for DNA libraries and sequenced with Illumina Novaseq 6000 PE150 mode. 46 and 48 lines were successfully sequenced and used in the final mutation analyses after we filtered out the lines that failed in library construction, genome sequencing (<15× depth of coverage), and/or cross-contamination (Table 2; Tables S1 to S6). All types of mutations were identified (Fig. 1).

**TABLE 2:** MA-line information on different strains of *S. pombe^a^*

Strains	NC	Lines	Depth	Divisions	BPSs	ts/tv	AT bias	In/Del	μ_BPS_	μ_Indel_	Data sources
ATCC-16979	0	46	142×	890	18	2.00	4.33	7.00	0.36	0.48	This study
ATCC-16979	4.0	48	69×	911	24	1.67	2.67	14.00	0.45	0.56	This study
972h-	0	79	25×	1952	696	0.72	1.68	6.13	1.70	1.74	Reference 58
ED668	0	96	62×	1716	395	0.78	1.57	6.00	2.00	0.60	Reference 32

^*a*^ AT bias, the mutation bias in the A/T direction; BPSs, total number of BPS mutations; depth, mean depth of sequencing coverage; divisions, mean cell divisions of each MA line; In/Del, ratio of insertion to deletion mutations; lines, number of MA lines per group; NC, norfloxacin concentration (μg/mL); ts/tv, ratio of transition to transversion mutations; μ_BPS_, BPS mutation rate (× 10^−10^); μ_Indel_, small-indel mutation rate (× 10^−10^).

Single-cell bottlenecking minimized selection during the MA process, but selection from both clonal expansion and norfloxacin treatment might bias the accumulated mutations. To test this, we calculated the ratio of nonsynonymous to synonymous substitutions of both the control and the norfloxacin treatment MA lines. There was no significant difference between the control (there are five non-synonymous and four synonymous substitutions; *χ*^2^ = 1.67, *d.f.* = 1, *P* = 0.20; Table S2) and the norfloxacin treatment MA lines (11 non-synonymous and two synonymous substitutions; *χ*^2^ = 0.03, *d.f.* = 1, *P* = 0.86; Table S5) in the ratios by taking into account the ratio of non-synonymous to synonymous sites (there are 6,074,090 non-synonymous and 1,641,376 synonymous sites in the genome) in ATCC-16979. There was also no significant difference between the two groups (*χ*^2^ = 1.04, *d.f.* = 1, *P* = 0.31). All the *χ*^2^ tests here were performed using Yates’ continuity correction (59). Moreover, we also estimated *Ne* during the MA process using a harmonic mean method (10.14 in the control and 10.39 in the treatment), suggesting that genetic drift dominated selection even upon norfloxacin treatment. Therefore, selection was minimal during norfloxacin-treated MA, which could thus be used for evaluating the mutagenic effects of norfloxacin on yeast.

**Fig 2:**
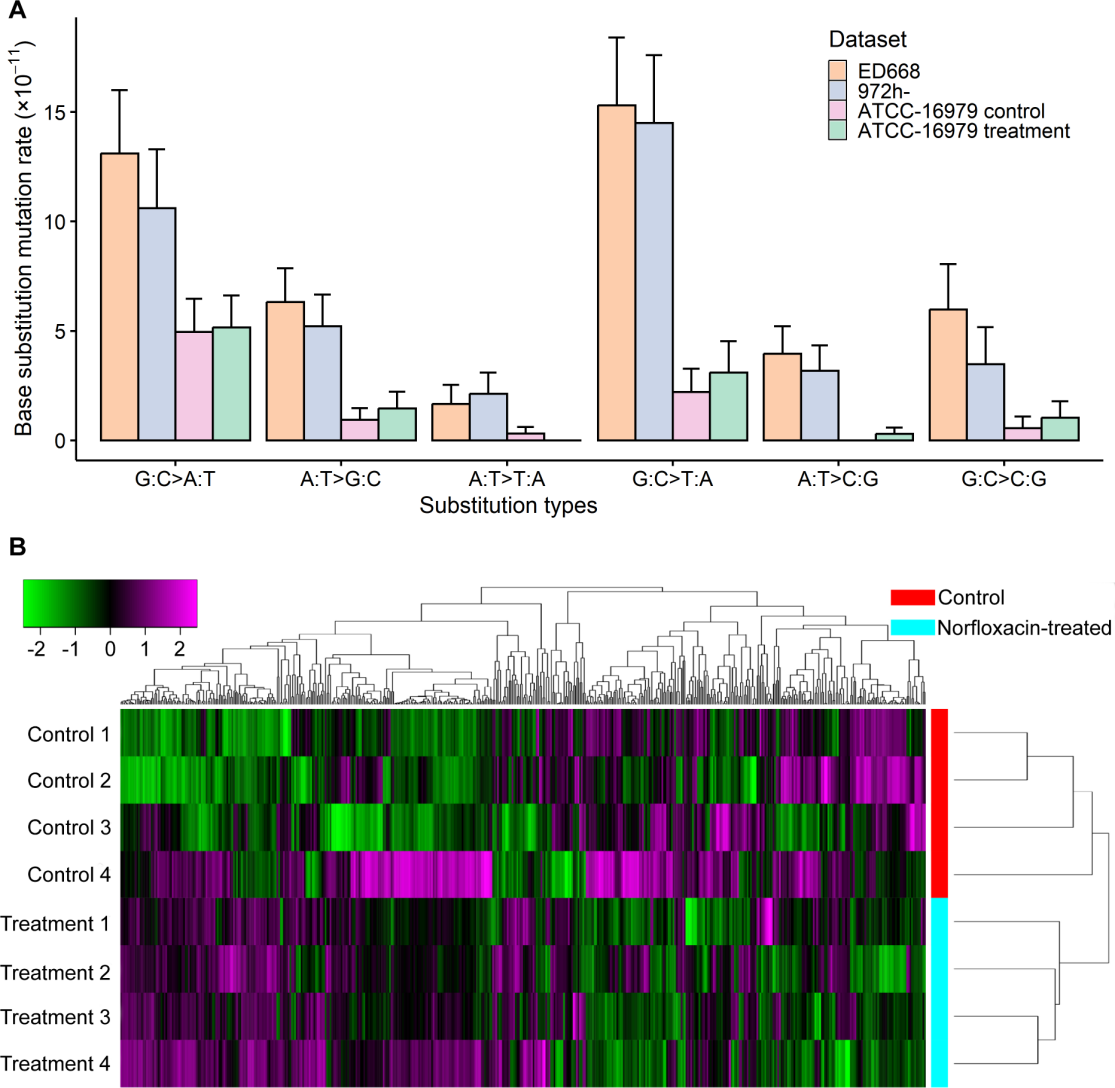
Mutation spectra and differential gene expression analysis. (**A**) Mutation spectra of the *S. pombe* ED668 (32), 972h- and ATCC-16979 control and norfloxacin-treated MA lines. The mutation spectrum of 972h- was estimated based on data from Behringer and Hall (58). Error bars denote SEM. (**B**) The heat map of differentially expressed genes in the control and the norfloxacin treatment.

For the control, we detected 18 BPSs from 46 MA lines with mean coverage of 142×, yielding a BPS mutation rate of 3.58 × 10^–11^ per nucleotide site per cell division (95% Poisson confidence intervals: 2.12 × 10^–11^, 5.66 × 10^–11^), which is not significantly different from the recently published spontaneous mutation rate of this strain (60), but the lowest among reported *S. pombe* mutation rates (Table 2). The transition/transversion ratio is 2.00, and the mutation bias in the A/T direction is 4.33 (Table 2; Table S1). In addition, we found 21 insertions and 3 deletions (size smaller than 50 bp), yielding an insertion/deletion ratio of 7.00 and an indel mutation rate of 4.77 × 10^–11^ (95% Poisson confidence intervals: 3.06 × 10^–11^, 7.10 × 10^–11^). This indel rate is not significantly different from the BPS rate, similar to the pattern reported in 972h- by Behringer and Hall (58) (Table 2; Table S3). Our results reveal an intriguing pattern where transitions are more common in ATCC-16979 than in the strains 972h- and ED668. Furthermore, ATCC-16979 has a high A/T bias of 4.33, but the difference among the three strains was not statistically significant (*χ*^2^ = 3.97, *d.f.* = 2, *P* = 0.137; Fig. 2A; Table 2).

48 MA lines in the norfloxacin treatment were sequenced at a mean depth of coverage 69× (ranging from 16× to 128×). We identified 24 BPSs across all 48 MA lines, yielding a BPS mutation rate of 4.47 × 10^–11^ per nucleotide site per cell division (95% Poisson confidence intervals: 2.86 × 10^–11^, 6.65 × 10^–11^), with a transition/transversion ratio of 1.67. Norfloxacin treatment thus did not significantly increase the mutation rate compared to the control (3.58 × 10^−11^, 95% CI: 2.12 × 10^−11^, 5.66 × 10^−11^; Poisson rate test, RR = 1.25, 95% CI: 0.68, 2.29, *P* = 0.47). The mutation bias in the A/T direction is 2.67 (Fig. 2A; Table 2; Table S4). We also detected 28 insertions and 2 deletions, leading to a small-indel mutation rate of 5.59 × 10^–11^ per site per cell division (CI: 3.77 × 10^–11^, 7.97 × 10^–11^; Table 2; Table S6). The lack of significant differences in the BPS and indel mutation rates between the control and the treatment MA lines suggests that a high concentration of norfloxacin does not affect the genomic mutation rate of *S. pombe*.

Structural variations (SVs) are critical for studying genome evolution, such as speciation and genome reduction (61, 62), especially in yeast (63–65). We detected SVs in the control and the treatment using Lumpy (v-0.2.13). Six and 13 SVs were detected in the control and treatment groups, respectively (each was visually checked with IGV; Tables S7 and S8). The SV rate is 1.19 × 10^–11^ (CI: 4.38 × 10^–12^, 2.60 × 10^–11^) in the control and 2.42 × 10^–11^ (CI: 1.29 × 10^–11^, 4.14 × 10^–11^) in the norfloxacin treatment. The results imply that the norfloxacin treatment does not significantly affect structural variations of *S. pombe*.

### Mutagenesis mechanisms of norfloxacin-treated cells revealed by RNAseq

After filtering out low-quality reads, a total of 284.49 and 299.64 million reads were obtained. The mean mapping rates for the control and the norfloxacin treatment were 95.56% and 95.84%, respectively. The RNAseq data of different replicate lines with high quality and repeatability were confirmed by principal component analysis and heat-map clustering analyses (Fig. 2B; Fig. S1).

**Fig 3:**
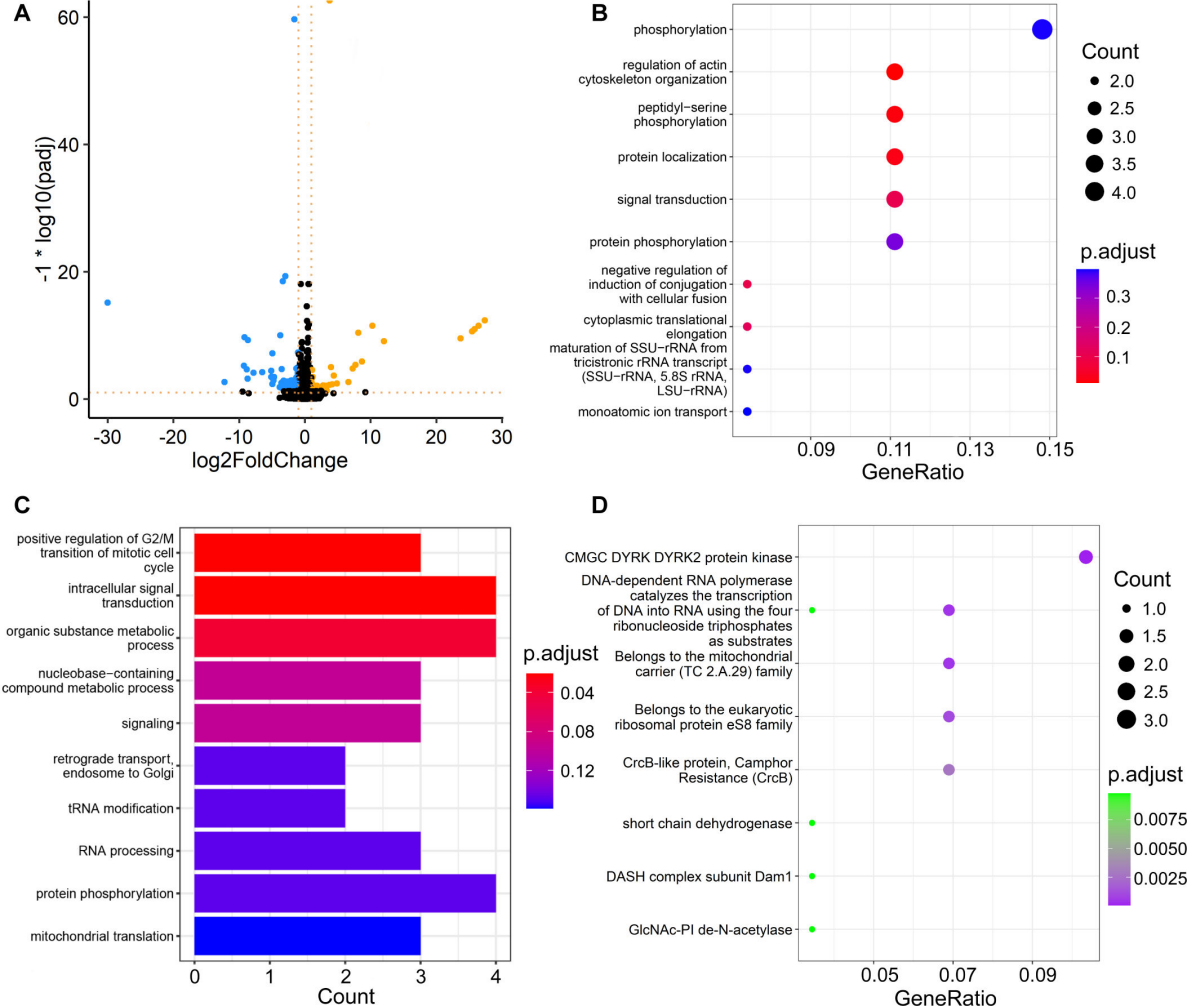
The differential gene expression of *S. pombe* ATCC-16979 upon norfloxacin treatment. (**A**) Differential gene expression upon norfloxacin treatment vs control. The dashed lines represent the threshold of extremely significantly up- or downregulated genes (|log_2_FoldChange| > 1 and *P*-adj ≤ 0.05). Yellow, blue, and black dots represent significantly upregulated, downregulated, and not significantly differentially expressed genes, respectively. (**B**) Upregulated genes in biological processes from GO analysis under norfloxacin treatment vs control. (**C**) Downregulated genes in biological processes from GO analysis under norfloxacin treatment vs control. (**D**) Upregulated genes in norfloxacin treatment vs control from the KEGG analysis.

Compared with the control, there are 74 downregulated (*P*-adj ≤ 0.05, log_2_FoldChange < −1) and 36 upregulated (*P*-adj ≤ 0.05, log_2_FoldChange > 1) genes in the norfloxacin treatment, including some with extreme significance and expression-level change (Fig. 3A). GO and KEGG analyses using the upregulated genes reveal that norfloxacin treatment on *S. pombe* elevates expression of pathways, such as metabolic kinase activity, cell division, and protein phosphorylation, as well as the nucleotide excision repair (NER) pathway (KEGG: K03017) of the cells (Fig. 3B and D; Figs. S2 to S4). The analyses on downregulated genes reveal that various catalytic reactions of metabolism dramatically decrease (Fig. 3C; Figs. S5 and S6), such as the formation of biofilms and signal transporters.

## DISCUSSION

Antibiotics have been the primary choice for disease treatment since their discovery. In recent years, the rise of bacterial resistance has intensified the fervor surrounding evolutionary research on antibiotics, such as the influence of norfloxacin on bacterial genomic mutation rate (10). Norfloxacin is known to elevate the bacterial genomic mutation rate by inhibiting the DNA gyrases and the topoisomerase IV of the DNA replication systems. This also raises the concern of whether it would also affect the genome stability of eukaryotic organisms at the DNA level, i.e., being mutagenic. If so, considering that some antibiotics can stay in the blood for days after they are injected into the patient’s body, such an effect is of concern for public health. In this study, we did not find any significant difference between the control and the norfloxacin-treated MA lines of *S. pombe* ATCC-16979, indicating that norfloxacin may not be mutagenic to eukaryotic genomes.

The NER pathway is one of the most widely distributed DNA repair processes in eukaryotes, in which damaged oligonucleotides are removed, followed by DNA synthesis to repair the lesions, ultimately leading to the rejoining of the resulting gaps (66–68). NER is able to repair a broad spectrum of lesions such as UV-induced DNA damage, chemically induced DNA monoadducts, and DNA-DNA crosslinks (69, 70). From our DEG results, NER was activated under norfloxacin treatment and might have alleviated the potential effects (the effects may not be strong) of norfloxacin. Based on the pathway-enrichment analyses, we did find that the NER pathway is actively expressed during norfloxacin treatment, via the upregulated gene *POLR2*. As mentioned earlier, there were no significant differences observed between the control and the treatment in terms of BPS, small indel, or structural variation mutation rates. We speculate that norfloxacin does not elevate the mutation rate of *S. pombe* because NER counteracts potential mutagenic effects, possibly from the elevated metabolism (many secondary metabolites are mutagenic). Other studies have also shown that NER plays an important role in repairing oxidative base damage, conferring resistance to methyl methanesulfonate, and repairing ultraviolet light damage in yeast (67, 71, 72), which may be consistent with the effects induced by our antibiotics. In humans, there is also a *POLR2* gene family in the genome, and the NER is the main pathway for removing helix-distorting DNA lesions and structures such as those formed by UV light, environmental mutagens, and some cancer chemotherapeutic adducts from DNA (66, 73). We speculate that norfloxacin causes eukaryotic DNA damage, while the NER detects and repairs most of them and thus averts an elevated mutation rate. NER demonstrates a robust capacity to repair damage when cells are exposed to such exogenous stressors. Investigating the potential impact of altered NER expression patterns on mutation rates under adverse growth conditions presents an intriguing question, warranting further exploration in future studies.

We recently demonstrated that the SOS response, an inducible response system induced by DNA damage, contributes to norfloxacin-induced mutation rate elevation in *E. coli* (10, 18). Over the past decades, there have been many studies showing that eukaryotic cells also have an inducible DNA repair capacity, functionally analogous to the SOS response in prokaryotic cells, to resist DNA damage and replication stress (74–77). For example, the *rad* and *rhp* genes are homologs of the *recA* gene in the SOS response (78–82). However, upon norfloxacin treatment, neither *rhp51* nor *rad* in *S. pombe* showed significant differential gene expression (*P*-adj ≤0.05, log_2_FoldChange < −1 or >1) (Table S9). This is again consistent with the non-elevated mutation rate of norfloxacin-treated *S. pombe* MA lines.

The limited number of mutations harvested in the control and norfloxacin-treated MA experiments might lead to low statistical power to detect a true difference when it exists. We thus conducted a simulation-based power analysis to evaluate the sensitivity of our experimental design. Using the actual experimental parameters (total sites × generations × lines in the control: g1 = 5.02822 × 10^11^, total sites × generations × lines in the treatment: g2 = 5.37022 × 10^11^, mutation rate in the control: μ_control = 3.58 × 10^–11^), we simulated mutation counts under a range of hypothetical effect sizes (multiplicative increase from 0% to 300%). For each effect size, 1,000 replicate data sets were generated and analyzed with a binomial test (α = 0.05). Power was defined as the proportion of simulations rejecting the null hypothesis. Results indicate that this study achieved 80% power to detect a minimum 100% increase (i.e., doubling) in mutation rate. This suggests that despite the modest mutation numbers, our study is adequately powered to detect significant differences in mutation rates between the norfloxacin-treated and control MA lines. Furthermore, even if we were to detect a significant difference between the two groups through additional sequencing or running the MA lines longer, the marginal increase observed in the treatment group still reinforces our conclusion that the mutagenic effects of norfloxacin treatment are negligible (3.58 × 10^–11^ for the control vs 4.47 × 10^–11^ for the treatment).

There are few studies on *S. pombe* ATCC-16979, mostly focusing on its ability to produce high yields of isobutanol, isoamyl alcohol, and ethanol (83, 84), and there was no reference genome available for *S. pombe* ATCC-16979. One of the most unexpected aspects of our study is the low spontaneous mutation rate of the strain used, as the BPS mutation rates in the other two strains of *S. pombe* (972h- and ED668) (32, 58) are 4.75× and 5.59× higher than that observed in the ATCC-16979 strain, respectively (Table 2). Maharjan and Ferenci (85) reported that BPS and 1 bp indel mutation rate significantly increased with decreasing growth rate in *E. coli* K12, i.e., the slower the growth rate, the higher the mutation rate. However, this cannot account for the much lower mutation rate of ATCC-16979 because its growth rate is the lowest, i.e., ~6.50 cell divisions per day vs 9.76 and 6.63 in 972h- and ED668, respectively (32, 58). It is not uncommon that the mutation rates can vary among different strains of the same species, but the underlying mechanisms remain entangled and unresolved (12, 86). Further research involving a broader range of strains is necessary to clarify the specific factors influencing the low mutation rate observed in ATCC-16979, as well as to determine the generalizability of norfloxacin’s non-mutagenic effects on *S. pombe*.

In summary, our study indicates that norfloxacin does not present a genotoxic hazard to eukaryotic and potentially human genomes, thus ensuring this pivotal antibiotic’s secure and responsible application in clinical settings. Nonetheless, heightened vigilance is warranted regarding the emergence of resistance mutations induced by norfloxacin in bacteria. These mutations can potentially escalate the risk of generating drug-resistant prokaryotic pathogens, consequently jeopardizing human health by impeding the efficacy of antibiotic therapies.

## Data Availability

All MA line raw sequences and RNAseq raw data were deposited in NCBI SRA with BioProject No. PRJNA961675. The *de novo* assembly and annotation of *S. pombe* ATCC-16979 were uploaded to National Genomics Data Center, China National Center for Bioinformation: GWHDEDB00000000.

## SUPPLEMENTAL MATERIAL

Supplemental figures spectrum.00233-25-s0001.docx Figs. S1 to S6.

Supplemental tables spectrum.00233-25-s0002.xlsx Tables S1 to S9.

ASM does not own the copyrights to Supplemental Material that may be linked to, or accessed through, an article. The authors have granted ASM a non-exclusive, world-wide license to publish the Supplemental Material files. Please contact the corresponding author directly for reuse.

## References

[1] Cabello FC. 2006. Heavy use of prophylactic antibiotics in aquaculture: a growing problem for human and animal health and for the environment. Environ Microbiol 8:1137–1144. doi:10.1111/j.1462-2920.2006.01054.x 16817922

[2] Kovalakova P, Cizmas L, McDonald TJ, Marsalek B, Feng M, Sharma VK. 2020. Occurrence and toxicity of antibiotics in the aquatic environment: a review. Chemosphere 251:126351. doi:10.1016/j.chemosphere.2020.126351 32443222

[3] Rohr JR, Barrett CB, Civitello DJ, Craft ME, Delius B, DeLeo GA, Hudson PJ, Jouanard N, Nguyen KH, Ostfeld RS, Remais JV, Riveau G, Sokolow SH, Tilman D. 2019. Emerging human infectious diseases and the links to global food production. Nat Sustain 2:445–456. doi:10.1038/s41893-019-0293-3 32219187 PMC7091874

[4] Le TX, Munekage Y, Kato S. 2005. Antibiotic resistance in bacteria from shrimp farming in mangrove areas. Sci Total Environ 349:95–105. doi:10.1016/j.scitotenv.2005.01.006 16198672

[5] Woodford N, Ellington MJ. 2007. The emergence of antibiotic resistance by mutation. Clin Microbiol Infect 13:5–18. doi:10.1111/j.1469-0691.2006.01492.x 17184282

[6] Miranda CD, Kehrenberg C, Ulep C, Schwarz S, Roberts MC. 2003. Diversity of tetracycline resistance genes in bacteria from Chilean salmon farms. Antimicrob Agents Chemother 47:883–888. doi:10.1128/AAC.47.3.883-888.2003 12604516 PMC149303

[7] Angulo FJ, Nargund VN, Chiller TC. 2004. Evidence of an association between use of anti-microbial agents in food animals and anti-microbial resistance among bacteria isolated from humans and the human health consequences of such resistance. J Vet Med Ser B 51:374–379. doi:10.1111/j.1439-0450.2004.00789.x 15525369

[8] Windels EM, Michiels JE, Fauvart M, Wenseleers T, Van den Bergh B, Michiels J. 2019. Bacterial persistence promotes the evolution of antibiotic resistance by increasing survival and mutation rates. ISME J 13:1239–1251. doi:10.1038/s41396-019-0344-9 30647458 PMC6474225

[9] Kohanski MA, DePristo MA, Collins JJ. 2010. Sublethal antibiotic treatment leads to multidrug resistance via radical-induced mutagenesis. Mol Cell 37:311–320. doi:10.1016/j.molcel.2010.01.003 20159551 PMC2840266

[10] Long H, Miller SF, Strauss C, Zhao C, Cheng L, Ye Z, Griffin K, Te R, Lee H, Chen C-C, Lynch M. 2016. Antibiotic treatment enhances the genome-wide mutation rate of target cells. Proc Natl Acad Sci USA 113:E2498–E2505. doi:10.1073/pnas.1601208113 27091991 PMC4983809

[11] Voolaid V, Jõers A, Kisand V, Tenson T. 2012. Co-occurrence of resistance to different antibiotics among aquatic bacteria. BMC Microbiol 12:1–8. doi:10.1186/1471-2180-12-225 23031674 PMC3519559

[12] Pan J, Li W, Ni J, Wu K, Konigsberg I, Rivera CE, Tincher C, Gregory C, Zhou X, Doak TG, Lee H, Wang Y, Gao X, Lynch M, Long H. 2022. Rates of mutations and transcript errors in the foodborne pathogen Salmonella enterica subsp. enterica. Mol Biol Evol 39:msac081. doi:10.1093/molbev/msac081 35446958 PMC9040049

[13] Jiang W, Lin T, Pan J, Rivera CE, Tincher C, Wang Y, Zhang Y, Gao X, Wang Y, Tsui H-CT, Winkler ME, Lynch M, Long H. 2024. Spontaneous mutations and mutational responses to penicillin treatment in the bacterial pathogen Streptococcus pneumoniae D39. Mar Life Sci Technol 6:198–211. doi:10.1007/s42995-024-00232-2 38827133 PMC11136922

[14] Lin T, Zhang J, Diao S, Yan J, Zhang K, Cao J, Huang J, Wang Y, Lv Z, Shen X, Sy SKB, Lynch M, Long H, Yu M. 2025. The impact of aztreonam-clavulanic acid exposure on gene expression and mutant selection using a multidrug-resistant E. coli. Microbiol Spectr 13:e0178224. doi:10.1128/spectrum.01782-24 39932309 PMC11878011

[15] Holmes B, Brogden RN, Richards DM. 1985. Norfloxacin: a review of its antibacterial activity, pharmacokinetic properties and therapeutic use. Drugs (Abingdon Engl) 30:482–513. doi:10.2165/00003495-198530060-00003 3908074

[16] Radman M. 1975. SOS repair hypothesis: phenomenology of an inducible DNA repair which is accompanied by mutagenesis. Basic Life Sci 5A:355–367. doi:10.1007/978-1-4684-2895-7_48 1103845

[17] Crumplin GC, Kenwright M, Hirst T. 1984. Investigations into the mechanism of action of the antibacterial agent norfloxacin. J Antimicrob Chemother 13 Suppl B:9–23. doi:10.1093/jac/13.suppl_b.9 6203890

[18] Lin T, Pan J, Gregory C, Wang Y, Tincher C, Rivera C, Lynch M, Long H, Zhang Y. 2023. Contribution of the SOS response and the DNA repair systems to norfloxacin induced mutations in E. coli. Mar Life Sci Technol 5:538–550. doi:10.1007/s42995-023-00185-y 38045542 PMC10689325

[19] Fernández J, Navasa M, Planas R, Montoliu S, Monfort D, Soriano G, Vila C, Pardo A, Quintero E, Vargas V, Such J, Ginès P, Arroyo V. 2007. Primary prophylaxis of spontaneous bacterial peritonitis delays hepatorenal syndrome and improves survival in cirrhosis. Gastroenterology 133:818–824. doi:10.1053/j.gastro.2007.06.065 17854593

[20] Zapater P, Caño R, Llanos L, Ruiz-Alcaraz AJ, Pascual S, Barquero C, Moreu R, Bellot P, Horga JF, Muñoz C, Pérez J, García-Peñarrubia P, Pérez-Mateo M, Such J, Francés R. 2009. Norfloxacin modulates the inflammatory response and directly affects neutrophils in patients with decompensated cirrhosis. Gastroenterology 137:1669–79. doi:10.1053/j.gastro.2009.07.058 19660462

[21] Moreau R, Elkrief L, Bureau C, Perarnau J-M, Thévenot T, Saliba F, Louvet A, Nahon P, Lannes A, Anty R, et al.. 2018. Effects of long-term norfloxacin therapy in patients with advanced cirrhosis. Gastroenterology 155:1816–1827. doi:10.1053/j.gastro.2018.08.026 30144431

[22] van Balen FA, Touw-Otten FW, de Melker RA. 1990. Single-dose pefloxacin versus five-days treatment with norfloxacin in uncomplicated cystitis in women. J Antimicrob Chemother 26 Suppl B:153–160. doi:10.1093/jac/26.suppl_b.153 2258342

[23] Corrado ML, Struble WE, Peter C, Hoagland V, Sabbaj J. 1987. Norfloxacin: review of safety studies. Am J Med 82:22–26. doi:10.1016/0002-9343(87)90614-0 3605158

[24] Swanson BN, Boppana VK, Vlasses PH, Rotmensch HH, Ferguson RK. 1983. Norfloxacin disposition after sequentially increasing oral doses. Antimicrob Agents Chemother 23:284–288. doi:10.1128/AAC.23.2.284 6220672 PMC186038

[25] Laurent JM, Garge RK, Teufel AI, Wilke CO, Kachroo AH, Marcotte EM. 2020. Humanization of yeast genes with multiple human orthologs reveals functional divergence between paralogs. PLoS Biol 18:e3000627. doi:10.1371/journal.pbio.3000627 32421706 PMC7259792

[26] Kachroo AH, Laurent JM, Yellman CM, Meyer AG, Wilke CO, Marcotte EM. 2015. Systematic humanization of yeast genes reveals conserved functions and genetic modularity. Science 348:921–925. doi:10.1126/science.aaa0769 25999509 PMC4718922

[27] Gachet Y, Codlin S, Hyams JS, Mole SE. 2005. btn1, the Schizosaccharomyces pombe homologue of the human Batten disease gene CLN3, regulates vacuole homeostasis. J Cell Sci 118:5525–5536. doi:10.1242/jcs.02656 16291725

[28] Volkmer E, Karnitz LM. 1999. Human homologs of Schizosaccharomyces pombe rad1, hus1, and rad9 form a DNA damage-responsive protein complex. J Biol Chem 274:567–570. doi:10.1074/jbc.274.2.567 9872989

[29] Wood V, Gwilliam R, Rajandream M-A, Lyne M, Lyne R, Stewart A, Sgouros J, Peat N, Hayles J, Baker S, et al.. 2002. The genome sequence of Schizosaccharomyces pombe. Nature 415:871–880. doi:10.1038/nature724 11859360

[30] Kibota TT, Lynch M. 1996. Estimate of the genomic mutation rate deleterious to overall fitness in E. coli. Nature 381:694–696. doi:10.1038/381694a0 8649513

[31] Pan J, Williams E, Sung W, Lynch M, Long H. 2021. The insect-killing bacterium Photorhabdus luminescens has the lowest mutation rate among bacteria. Mar Life Sci Technol 3:20–27. doi:10.1007/s42995-020-00060-0 33791681 PMC8009600

[32] Farlow A, Long H, Arnoux S, Sung W, Doak TG, Nordborg M, Lynch M. 2015. The spontaneous mutation rate in the fission yeast Schizosaccharomyces pombe. Genetics 201:737–744. doi:10.1534/genetics.115.177329 26265703 PMC4596680

[33] Wahl LM, Gerrish PJ. 2001. The probability that beneficial mutations are lost in populations with periodic bottlenecks. Evolution (N Y) 55:2606–2610. doi:10.1111/j.0014-3820.2001.tb00772.x 11831673

[34] Li H, Wu K, Ruan C, Pan J, Wang Y, Long H. 2019. Cost-reduction strategies in massive genomics experiments. Mar Life Sci Technol 1:15–21. doi:10.1007/s42995-019-00013-2

[35] De Coster W, D’Hert S, Schultz DT, Cruts M, Van Broeckhoven C. 2018. NanoPack: visualizing and processing long-read sequencing data. Bioinformatics 34:2666–2669. doi:10.1093/bioinformatics/bty149 29547981 PMC6061794

[36] Kolmogorov M, Yuan J, Lin Y, Pevzner PA. 2019. Assembly of long, error-prone reads using repeat graphs. Nat Biotechnol 37:540–546. doi:10.1038/s41587-019-0072-8 30936562

[37] Walker BJ, Abeel T, Shea T, Priest M, Abouelliel A, Sakthikumar S, Cuomo CA, Zeng Q, Wortman J, Young SK, Earl AM. 2014. Pilon: an integrated tool for comprehensive microbial variant detection and genome assembly improvement. PLoS One 9:e112963. doi:10.1371/journal.pone.0112963 25409509 PMC4237348

[38] Mikheenko A, Prjibelski A, Saveliev V, Antipov D, Gurevich A. 2018. Versatile genome assembly evaluation with QUAST-LG. Bioinformatics 34:i142–i150. doi:10.1093/bioinformatics/bty266 29949969 PMC6022658

[39] Simão FA, Waterhouse RM, Ioannidis P, Kriventseva EV, Zdobnov EM. 2015. BUSCO: assessing genome assembly and annotation completeness with single-copy orthologs. Bioinformatics 31:3210–3212. doi:10.1093/bioinformatics/btv351 26059717

[40] Grabherr MG, Haas BJ, Yassour M, Levin JZ, Thompson DA, Amit I, Adiconis X, Fan L, Raychowdhury R, Zeng Q, Chen Z, Mauceli E, Hacohen N, Gnirke A, Rhind N, di Palma F, Birren BW, Nusbaum C, Lindblad-Toh K, Friedman N, Regev A. 2011. Full-length transcriptome assembly from RNA-Seq data without a reference genome. Nat Biotechnol 29:644–652. doi:10.1038/nbt.1883 21572440 PMC3571712

[41] Stanke M, Morgenstern B. 2005. AUGUSTUS: a web server for gene prediction in eukaryotes that allows user-defined constraints. Nucleic Acids Res 33:W465–W467. doi:10.1093/nar/gki458 15980513 PMC1160219

[42] Brůna T, Hoff KJ, Lomsadze A, Stanke M, Borodovsky M. 2021. BRAKER2: automatic eukaryotic genome annotation with GeneMark-EP+ and AUGUSTUS supported by a protein database. NAR Genom Bioinform 3:lqaa108. doi:10.1093/nargab/lqaa108 33575650 PMC7787252

[43] Götz S, García-Gómez JM, Terol J, Williams TD, Nagaraj SH, Nueda MJ, Robles M, Talón M, Dopazo J, Conesa A. 2008. High-throughput functional annotation and data mining with the Blast2GO suite. Nucleic Acids Res 36:3420–3435. doi:10.1093/nar/gkn176 18445632 PMC2425479

[44] Lowe TM, Chan PP. 2016. tRNAscan-SE On-line: integrating search and context for analysis of transfer RNA genes. Nucleic Acids Res 44:W54–W57. doi:10.1093/nar/gkw413 27174935 PMC4987944

[45] Bolger AM, Lohse M, Usadel B. 2014. Trimmomatic: a flexible trimmer for Illumina sequence data. Bioinformatics 30:2114–2120. doi:10.1093/bioinformatics/btu170 24695404 PMC4103590

[46] Li H, Durbin R. 2009. Fast and accurate short read alignment with Burrows-Wheeler transform. Bioinformatics 25:1754–1760. doi:10.1093/bioinformatics/btp324 19451168 PMC2705234

[47] McKenna A, Hanna M, Banks E, Sivachenko A, Cibulskis K, Kernytsky A, Garimella K, Altshuler D, Gabriel S, Daly M, DePristo MA. 2010. The Genome Analysis Toolkit: a MapReduce framework for analyzing next-generation DNA sequencing data. Genome Res 20:1297–1303. doi:10.1101/gr.107524.110 20644199 PMC2928508

[48] DePristo MA, Banks E, Poplin R, Garimella KV, Maguire JR, Hartl C, Philippakis AA, del Angel G, Rivas MA, Hanna M, McKenna A, Fennell TJ, Kernytsky AM, Sivachenko AY, Cibulskis K, Gabriel SB, Altshuler D, Daly MJ. 2011. A framework for variation discovery and genotyping using next-generation DNA sequencing data. Nat Genet 43:491–498. doi:10.1038/ng.806 21478889 PMC3083463

[49] Thorvaldsdottir H, Robinson JT, Mesirov JP. 2013. Integrative Genomics Viewer (IGV): high-performance genomics data visualization and exploration. Brief Bioinformatics 14:178–192. doi:10.1093/bib/bbs017 22517427 PMC3603213

[50] R core team. 2013. R: a language and environment for statistical computing. R Foundation for Statistical Computing, Vienna, Austria.

[51] Chen S, Zhou Y, Chen Y, Gu J. 2018. fastp: an ultra-fast all-in-one FASTQ preprocessor. Bioinformatics 34:i884–i890. doi:10.1093/bioinformatics/bty560 30423086 PMC6129281

[52] Kim D, Paggi JM, Park C, Bennett C, Salzberg SL. 2019. Graph-based genome alignment and genotyping with HISAT2 and HISAT-genotype. Nat Biotechnol 37:907–915. doi:10.1038/s41587-019-0201-4 31375807 PMC7605509

[53] Danecek P, Bonfield JK, Liddle J, Marshall J, Ohan V, Pollard MO, Whitwham A, Keane T, McCarthy SA, Davies RM, Li H. 2021. Twelve years of SAMtools and BCFtools. Gigascience 10:giab008. doi:10.1093/gigascience/giab008 33590861 PMC7931819

[54] Pertea M, Pertea GM, Antonescu CM, Chang T-C, Mendell JT, Salzberg SL. 2015. StringTie enables improved reconstruction of a transcriptome from RNA-seq reads. Nat Biotechnol 33:290–295. doi:10.1038/nbt.3122 25690850 PMC4643835

[55] Love MI, Huber W, Anders S. 2014. Moderated estimation of fold change and dispersion for RNA-seq data with DESeq2. Genome Biol 15:550. doi:10.1186/s13059-014-0550-8 25516281 PMC4302049

[56] Yu G, Wang L-G, Han Y, He Q-Y. 2012. clusterProfiler: an R package for comparing biological themes among gene clusters. OMICS J Integr Biol 16:284–287. doi:10.1089/omi.2011.0118 PMC3339379 22455463

[57] Huang DW, Sherman BT, Tan Q, Kir J, Liu D, Bryant D, Guo Y, Stephens R, Baseler MW, Lane HC, Lempicki RA. 2007. DAVID Bioinformatics Resources: expanded annotation database and novel algorithms to better extract biology from large gene lists. Nucleic Acids Res 35:W169–W175. doi:10.1093/nar/gkm415 17576678 PMC1933169

[58] Behringer MG, Hall DW. 2016. Genome-wide estimates of mutation rates and spectrum in Schizosaccharomyces pombe indicate CpG sites are highly mutagenic despite the absence of DNA methylation. G3 6:149–160. doi:10.1534/g3.115.022129 PMC4704713 26564949

[59] Yates F. 1984. Tests of significance for 2 × 2 contingency tables. J R Stat Soc Ser A 147:426. doi:10.2307/2981577

[60] Wu K, Li H, Wang Y, Liu D, Li H, Zhang Y, Lynch M, Long H. 2023. Silver nanoparticles elevate mutagenesis of eukaryotic genomes. G3 (Bethesda) 13:jkad008. doi:10.1093/g3journal/jkad008 36635051 PMC9997555

[61] Sloan DB, Moran NA. 2013. The evolution of genomic instability in the obligate endosymbionts of whiteflies. Genome Biol Evol 5:783–793. doi:10.1093/gbe/evt044 23542079 PMC3673631

[62] Delneri D, Colson I, Grammenoudi S, Roberts IN, Louis EJ, Oliver SG. 2003. Engineering evolution to study speciation in yeasts. Nature 422:68–72. doi:10.1038/nature01418 12621434

[63] Zhang K, Zhang L-J, Fang Y-H, Jin X-N, Qi L, Wu X-C, Zheng D-Q. 2016. Genomic structural variation contributes to phenotypic change of industrial bioethanol yeast Saccharomyces cerevisiae. FEMS Yeast Res 16:fov118. doi:10.1093/femsyr/fov118 26733503

[64] Chen S, Xie Z-X, Yuan Y-J. 2020. Discovering and genotyping genomic structural variations by yeast genome synthesis and inducible evolution. FEMS Yeast Res 20:foaa012. doi:10.1093/femsyr/foaa012 32188997

[65] Jeffares DC, Jolly C, Hoti M, Speed D, Shaw L, Rallis C, Balloux F, Dessimoz C, Bähler J, Sedlazeck FJ. 2017. Transient structural variations have strong effects on quantitative traits and reproductive isolation in fission yeast. Nat Commun 8:14061. doi:10.1038/ncomms14061 28117401 PMC5286201

[66] de Laat WL, Jaspers NG, Hoeijmakers JH. 1999. Molecular mechanism of nucleotide excision repair. Genes Dev 13:768–785. doi:10.1101/gad.13.7.768 10197977

[67] Memisoglu A, Samson L. 2000. Contribution of base excision repair, nucleotide excision repair, and DNA recombination to alkylation resistance of the fission yeast Schizosaccharomyces pombe. J Bacteriol 182:2104–2112. doi:10.1128/JB.182.8.2104-2112.2000 10735851 PMC111257

[68] Huang J-C, Hsu DS, Kazantsev A, Sancar A. 1994. Substrate spectrum of human excinuclease: repair of abasic sites, methylated bases, mismatches, and bulky adducts. Proc Natl Acad Sci USA 91:12213–12217. doi:10.1073/pnas.91.25.12213 7991608 PMC45407

[69] Mu D, Tursun M, Duckett DR, Drummond JT, Modrich P, Sancar A. 1997. Recognition and repair of compound DNA lesions (base damage and mismatch) by human mismatch repair and excision repair systems. Mol Cell Biol 17:760–769. doi:10.1128/MCB.17.2.760 9001230 PMC231802

[70] Buschta-Hedayat N, Buterin T, Hess MT, Missura M, Naegeli H. 1999. Recognition of nonhybridizing base pairs during nucleotide excision repair of DNA. Proc Natl Acad Sci USA 96:6090–6095. doi:10.1073/pnas.96.11.6090 10339546 PMC26840

[71] Prakash S, Prakash L. 2000. Nucleotide excision repair in yeast. Mutat Res 451:13–24. doi:10.1016/s0027-5107(00)00037-3 10915862

[72] Scott AD, Neishabury M, Jones DH, Reed SH, Boiteux S, Waters R. 1999. Spontaneous mutation, oxidative DNA damage, and the roles of base and nucleotide excision repair in the yeast Saccharomyces cerevisiae. Yeast 15:205–218. doi:10.1002/(SICI)1097-0061(199902)15:3<205::AID-YEA361>3.0.CO;2-1 10077187

[73] Muñoz JC, Beckerman I, Choudhary R, Bouvier LA, Muñoz MJ. 2022. DNA damage-induced RNAPII degradation and its consequences in gene expression. Genes (Basel) 13:1951. doi:10.3390/genes13111951 36360188 PMC9689695

[74] Eller MS, Maeda T, Magnoni C, Atwal D, Gilchrest BA. 1997. Enhancement of DNA repair in human skin cells by thymidine dinucleotides: evidence for a p53-mediated mammalian SOS response. Proc Natl Acad Sci USA 94:12627–12632. doi:10.1073/pnas.94.23.12627 9356500 PMC25061

[75] Eller MS, Asarch A, Gilchrest BA. 2008. Photoprotection in human skin—a multifaceted SOS response. Photochem Photobiol 84:339–349. doi:10.1111/j.1751-1097.2007.00264.x 18179622

[76] Fu Y, Zhu Y, Zhang K, Yeung M, Durocher D, Xiao W. 2008. Rad6-Rad18 mediates a eukaryotic SOS response by ubiquitinating the 9-1-1 checkpoint clamp. Cell 133:601–611. doi:10.1016/j.cell.2008.02.050 18485869

[77] Eller MS, Gilchrest BA. 2000. Tanning as part of the eukaryotic SOS response. Pigment Cell Res 13:94–97. doi:10.1034/j.1600-0749.13.s8.17.x 11041364

[78] Jang YK, Jin YH, Kim EM, Fabre F, Hong SH, Park SD. 1994. Cloning and sequence analysis of rhp51^+^, a Schizosaccharomyces pombe homolog of the Saccharomyces cerevisiae RAD51 gene. Gene 142:207–211. doi:10.1016/0378-1119(94)90262-3 8194753

[79] Muris DF, Vreeken K, Carr AM, Broughton BC, Lehmann AR, Lohman PH, Pastink A. 1993. Cloning the RAD51 homologue of Schizosaccharomyces pombe. Nucleic Acids Res 21:4586–4591. doi:10.1093/nar/21.19.4586 8233794 PMC311194

[80] Aboussekhra A, Chanet R, Adjiri A, Fabre F. 1992. Semidominant suppressors of Srs2 helicase mutations of Saccharomyces cerevisiae map in the RAD51 gene, whose sequence predicts a protein with similarities to procaryotic RecA proteins. Mol Cell Biol 12:3224–3234. doi:10.1128/mcb.12.7.3224-3234.1992 1620127 PMC364537

[81] Basile G, Aker M, Mortimer RK. 1992. Nucleotide sequence and transcriptional regulation of the yeast recombinational repair gene RAD51. Mol Cell Biol 12:3235–3246. doi:10.1128/mcb.12.7.3235-3246.1992 1620128 PMC364538

[82] Shinohara A, Ogawa H, Ogawa T. 1992. Rad51 protein involved in repair and recombination in S. cerevisiae is a RecA-like protein. Cell 69:457–470. doi:10.1016/0092-8674(92)90447-k 1581961

[83] Morneau AD, Zuehlke JM, Edwards CG. 2011. Comparison of media formulations used to selectively cultivate Dekkera/Brettanomyces. Lett Appl Microbiol 53:460–465. doi:10.1111/j.1472-765X.2011.03133.x 21812795

[84] Wu Q, Xu Y, Chen L. 2012. Diversity of yeast species during fermentative process contributing to Chinese Maotai-flavour liquor making. Lett Appl Microbiol 55(4):301–307. doi:10.1111/j.1472-765X.2012.03294.x 22862564

[85] Maharjan RP, Ferenci T. 2018. The impact of growth rate and environmental factors on mutation rates and spectra in Escherichia coli. Environ Microbiol Rep 10:626–633. doi:10.1111/1758-2229.12661 29797781

[86] Ness RW, Morgan AD, Vasanthakrishnan RB, Colegrave N, Keightley PD. 2015. Extensive de novo mutation rate variation between individuals and across the genome of Chlamydomonas reinhardtii. Genome Res 25:1739–1749. doi:10.1101/gr.191494.115 26260971 PMC4617969

