# Supplementary material for: The negligible mutagenic effects of norfloxacin on the genome of the fission yeast Schizosaccharomyces pombe ATCC-16979
Source: Microbiol Spectr. 2025 Jul 25;13(9):e00233-25. doi: 10.1128/spectrum.00233-25 (PMC12403669; doi:10.1128/spectrum.00233-25)
Supplement: Supplemental figures — Figs. S1 to S6. [file spectrum.00233-25-s0001.docx]

**Figure Legends**

**
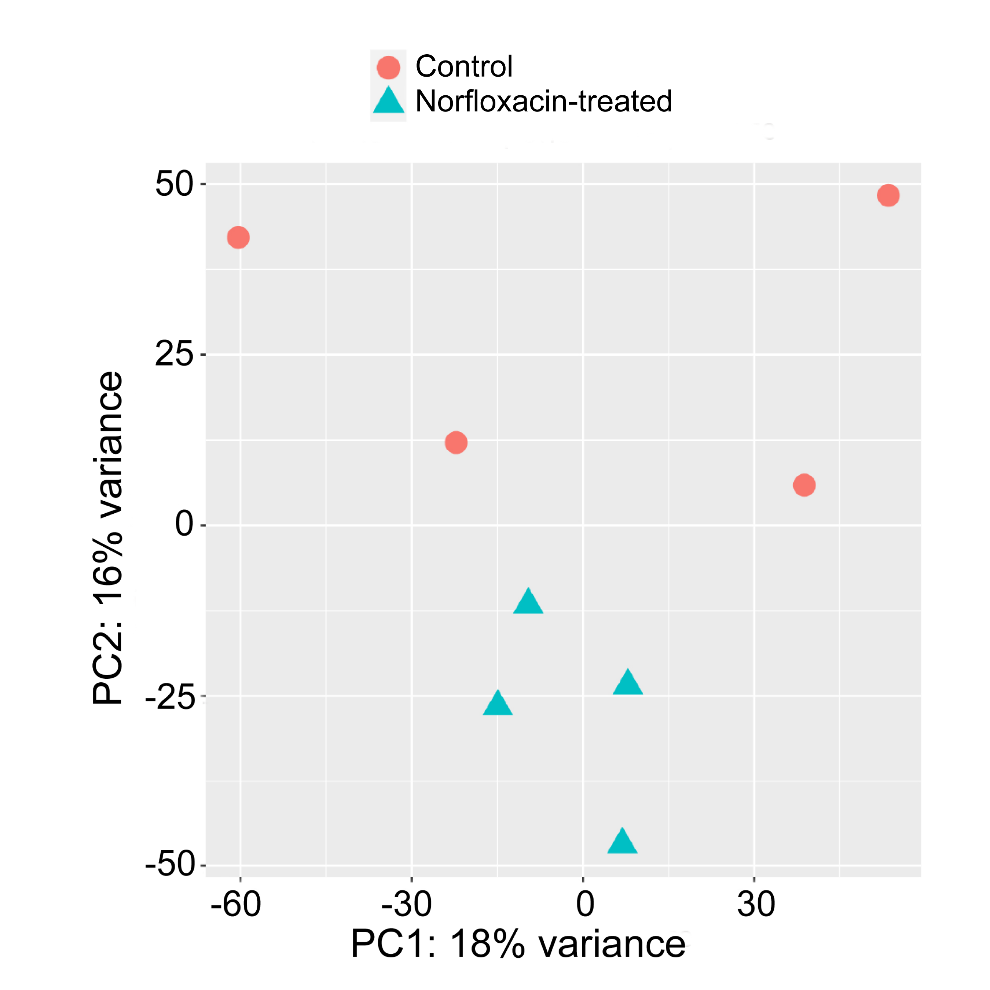
**

**Supplementary Fig. S1.** The PCA plot, based on gene expression from RNAseq.


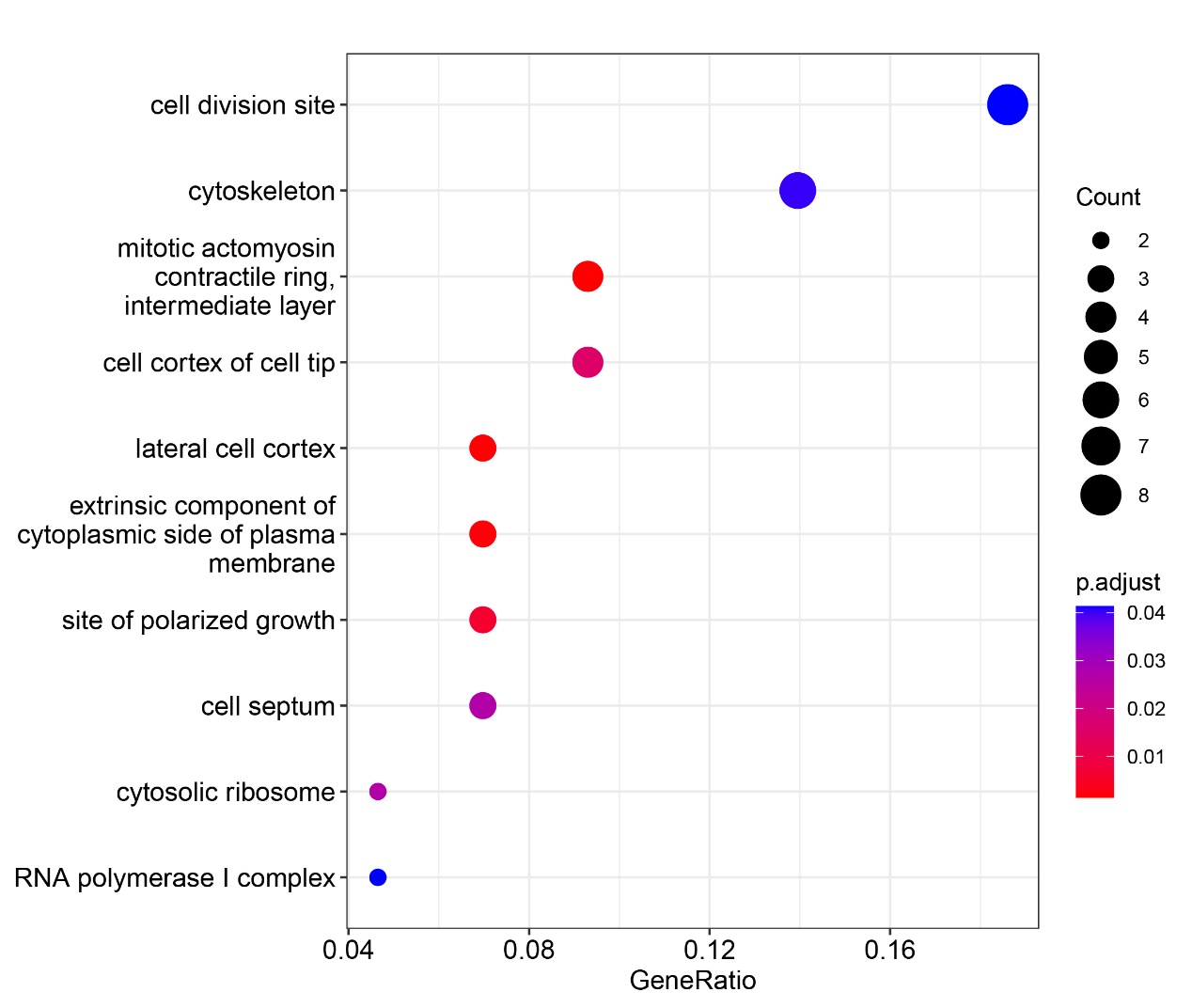


**Supplementary Fig. S2.** Up-regulated genes in cellular components from GO analysis (norfloxacin treatment vs control).


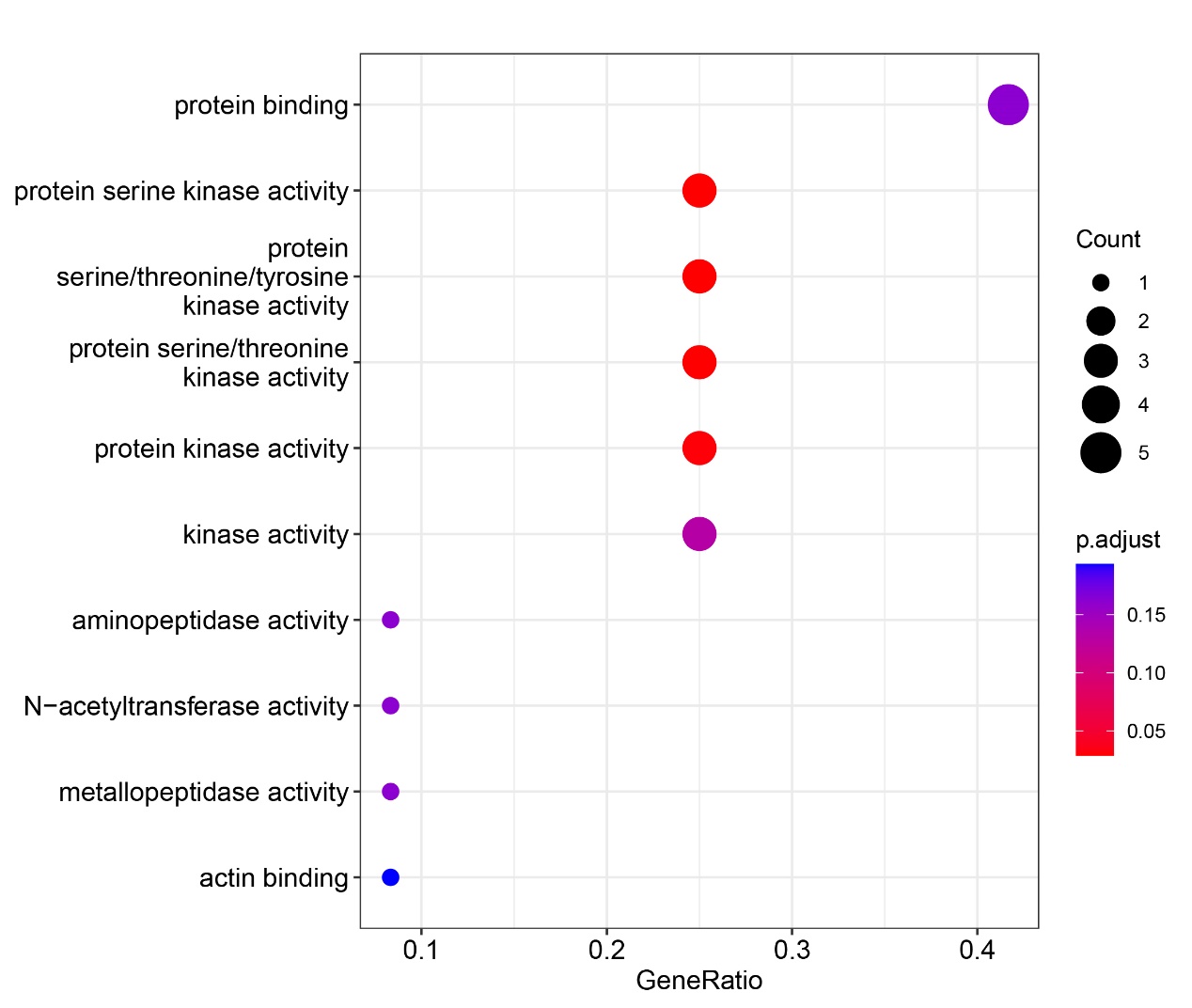


**Supplementary Fig. S3.** Up-regulated genes in molecular function from GO analysis (norfloxacin treatment vs control).


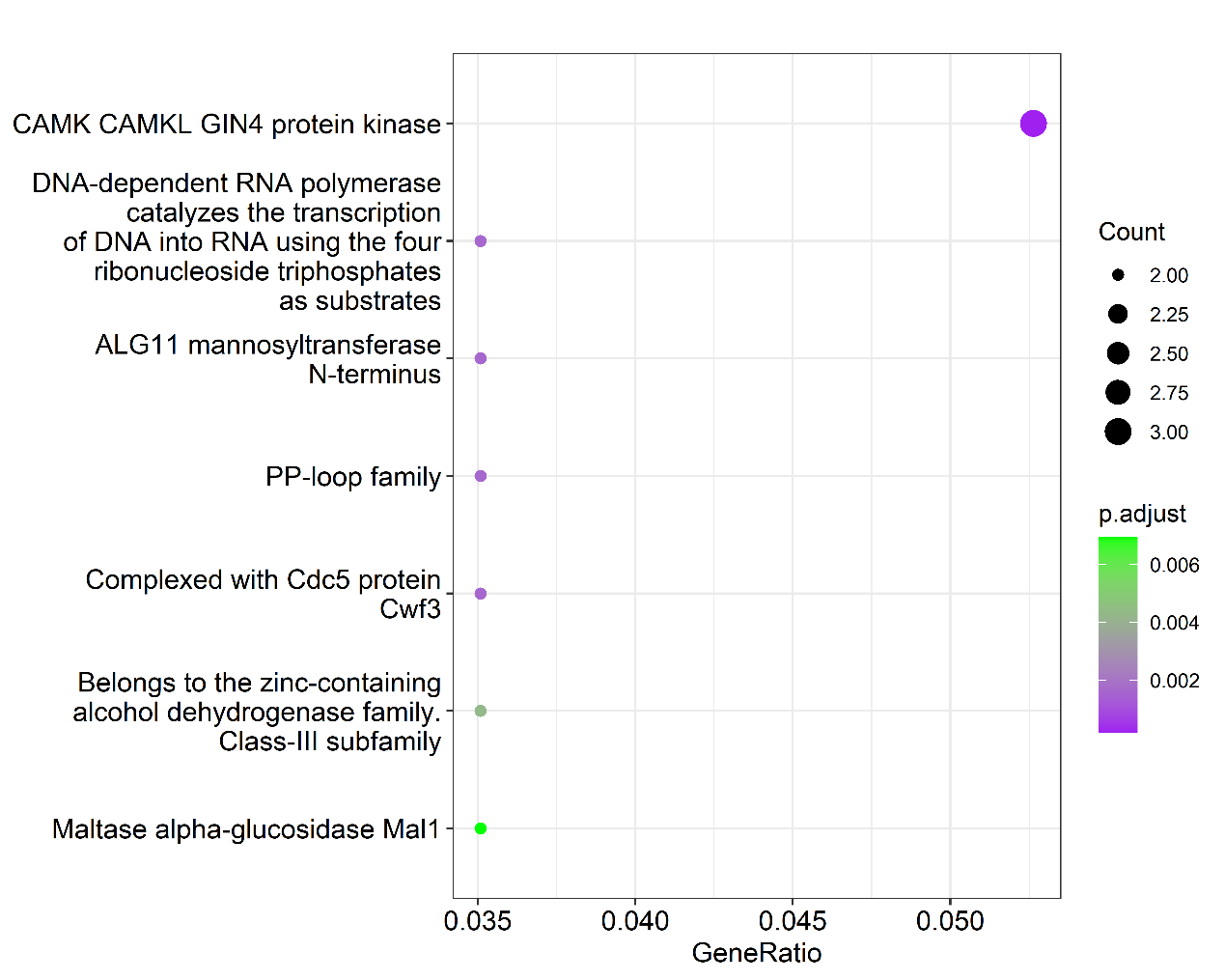


**Supplementary Fig. S4.** Down-regulated genes from KEGG analysis (norfloxacin treatment vs control).


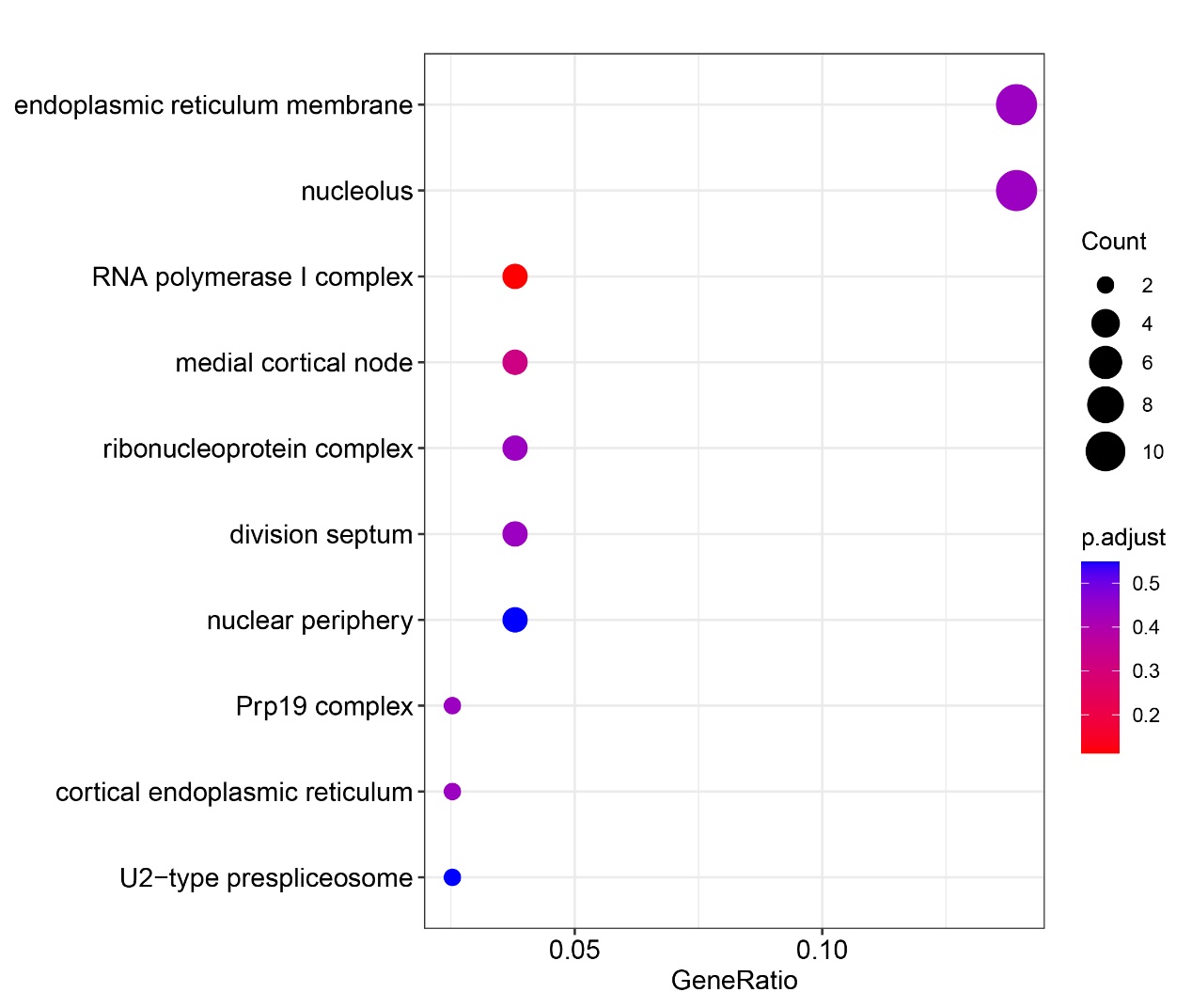


**Supplementary Fig. S5.** Down-regulated genes in cellular component from GO analysis (norfloxacin treatment vs control).


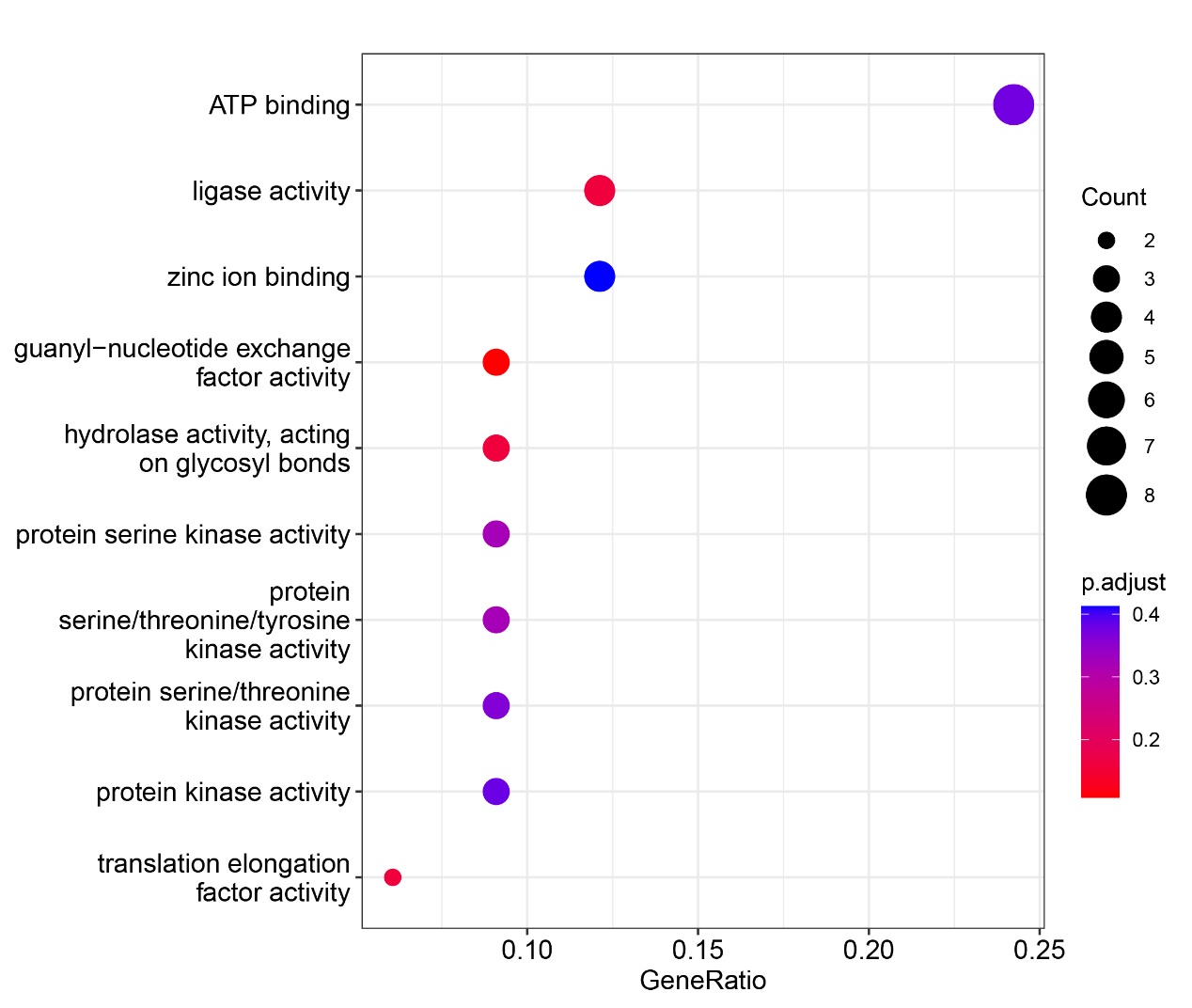


**Supplementary Fig. S6.** Down-regulated genes in molecular function from GO analysis (norfloxacin treatment vs control).
